# Detection of* Babesia caballi* and* Theileria equi* in Blood from Equines from Four Indigenous Communities in Costa Rica

**DOI:** 10.1155/2015/236278

**Published:** 2015-11-16

**Authors:** María Fernanda Posada-Guzmán, Gaby Dolz, Juan José Romero-Zúñiga, Ana Eugenia Jiménez-Rocha

**Affiliations:** ^1^Maestría en Enfermedades Tropicales, Posgrado Regional en Ciencias Veterinarias Tropicales, Universidad Nacional, Campus Presbítero Benjamín Nuñez, P.O. Box 86, 3000 Heredia, Costa Rica; ^2^Programa de Investigación en Medicina Poblacional, Escuela de Medicina Veterinaria, Universidad Nacional, Campus Presbítero Benjamín Nuñez, P.O. Box 86, 3000 Heredia, Costa Rica; ^3^Laboratorio de Parasitología, Escuela de Medicina Veterinaria, Universidad Nacional, Campus Presbítero Benjamín Nuñez, P.O. Box 86, 3000 Heredia, Costa Rica

## Abstract

A cross-sectional study was carried out in four indigenous communities of Costa Rica to detect presence and prevalence of* Babesia caballi *and* Theileria equi* and to investigate factors associated with presence of these hemoparasites. General condition of horses (*n* = 285) was evaluated, and hematocrits and hemoglobin were determined from blood samples of 130 horses, which were also analyzed using blood smears, nested polymerase chain reaction (PCR), and immunosorbent assay (c-ELISA). The general condition of the horses (*n* = 285) in terms of their body and coat was between regular and poor, and hematocrit and hemoglobin average values were low (19% and 10.65 g/dL, resp.). Erythrocyte inclusions were observed in 32 (24.6%) of the samples. Twenty-six samples (20.0%) gave positive results for* B. caballi* and 60 (46.2%) for* T. equi*; 10 horses (7.7%) showed mixed infection, when analyzed by PCR. Using c-ELISA, it was found that 90 (69.2%) horses had antibodies against* B. caballi* and 115 (88.5%) against* T. equi*, while 81 (62.3%) showed mixed reactions. There were no factors associated with the presence of* B. caballi* and* T. equi*. These results contrast with results previously obtained in equines in the Central Valley of Costa Rica.

## 1. Introduction

Equine piroplasmosis is a horse infection produced by* Babesia caballi *and* Theileria equi *protozoan that belong, respectively, to the Babesiidae and Theileriidae families of the order Piroplasmida. The two agents may infest an animal at the same time and they are transmitted by ticks [1, 2].

The parasites that cause equine piroplasmosis are endemic in most parts of the world, including America. Seroprevalence between 20% and 90% is reported in Latin America, where the prevalence of* T. equi* is greater than that of* B. caballi*, while in regions of the Northern Hemisphere a greater prevalence of* B. caballi* than of* T. equi* has been observed; in addition, mixed infections were reported close to 30% [3–6].

Piroplasmosis is difficult to diagnose in endemic regions where clinical signs are variable rather than specific [7, 8].* T. equi* is considered to be the most pathogenic, able to generate fevers of up to 40°C, lymphadenopathy, hepatomegaly, and bilirubinuria [9, 10].

Causative agents of equine piroplasmosis can be observed under a microscope through Giemsa-stained blood smears; however, this technique has the disadvantage of being subjective and having low sensitivity, mostly in horses with low parasitemia (<10^4^ infected erythrocytes/*μ*L) [6, 11, 12]. Indirect immunofluorescence assay (IFA) and immunosorbent assays (ELISA) are the serological tests required by the World Organization on Animal Health (OIE) for horses that have to be transported from one country to another [2]. Animals that are persistently infected or with an active infection will show high antibody titers, while, in horses that eliminate the agent, antibodies will decrease to the point where they cannot be detected in a period of six to ten months [13].

The polymerase chain reaction (PCR) technique for* B. caballi* and* T. equi* is highly efficient, since it can detect low levels of parasitemia, determines the infecting species, and also allows the detection of persistently infected animals and chronic carriers [14–17]. However, time and cost of implementation are two inconvenient aspects of this technique.

The different species of tick genera involved in the transmission of equine piroplasmosis,* Amblyomma*,* Dermacentor*, and* Rhipicephalus*, have been reported in the country by Álvarez et al. [18]. Until now, four serologic studies have been carried out in Costa Rica, detecting the presence of antibodies against* B. caballi* and* T. equi* in equines participating in a rally (*n* = 41) [19], for exportation purposes (*n* = 21) [20], from slaughterhouses (*n* = 51) [21], and also from thoroughbred stabled equines (*n* = 181) from the Central Valley [22], where the prevalence of 44.3% to 59.0% for* B. caballi* and of 34.0% to 47.0% for* T. equi* was determined.

The situation of piroplasmosis in indigenous horses in these communities has not been studied, even though these animals provide source of subsistence to their owners, and effective parasite control is lacking [3]. In contrast, most horses of the rest of the country are tested and dewormed regularly by their owners, especially when they are housed in stables or to participate in competitions. The objective of the present study was to determine the presence of current and past infections of* B. caballi *and* T. equi* in blood samples in horses of indigenous communities and determine risk factors associated with the presence of these agents.

## 2. Materials and Methods

### 2.1. Study Type and Reference Population

**Figure 1:**
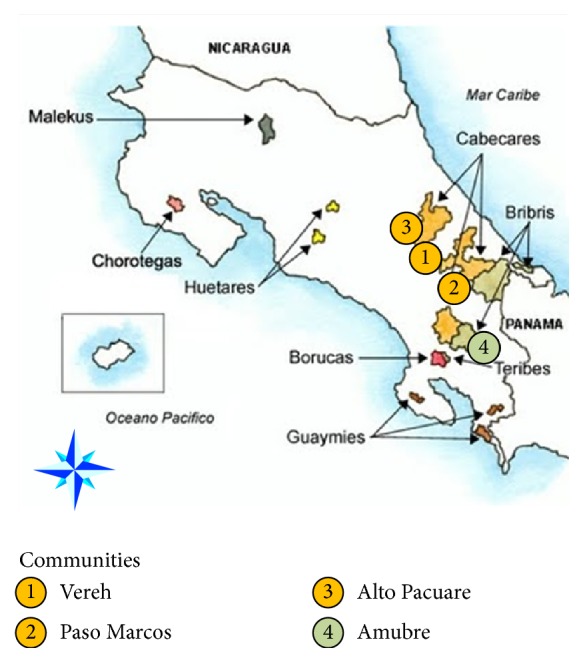
Location of indigenous communities in Costa Rica, showing the four sites sampled (taken from http://costarica1.bligoo.com.mx/?page=2 and modified).

A descriptive cross-sectional observational study was carried out between March and October 2011 in the communities of Vereh, Paso Marcos, Alto Pacuare, and Amubre (Figure 1). The first three communities are in the Cabecar reserve, in Chirripó, Turrialba (9°41′45.81 to 9°48′46.12 North and 83°25′51.90 to 83°29′21.50 West), while the Amubre community is in Talamanca and belongs to the Bribri group (9°30′ North and 83°40′ West). Each community was visited once.

### 2.2. Data and Sample Collection

A survey with closed answers was conducted with each owner of an animal regarding the origin and general health care of the horse. Then a general clinical exam was carried out, and blood samples were taken. On an individual clinical record the presence of clinical signs was recorded (anemia, weakness, anorexia, cachexia, and hemoglobinuria), as well as the horse's body condition [23], coat condition, and tick presence.

Blood samples from all horses in work places were collected. The samples were transported under refrigeration to the laboratory, where clinical analysis and blood smear were carried out; samples were finally stored at −20°C for their molecular analysis. Blood samples without EDTA were centrifuged for 10 minutes at 10000 g and stored at −20°C for serological analysis.

The total number of samples collected in each community is shown in Table 1. Of the 285 samples taken in the field, 45% were selected from each community (for a total of 130 horses). The samples were selected on the basis of convenience, that is, the most affected were chosen over the least affected (horses presenting clinical signs or having ticks on them), and tested for erythrocyte inclusions and by PCR and ELISA.

### 2.3. Clinical Analyses and Blood Smears

Hematocrit values were determined for 130 samples in a HETTICH microcentrifuge (18600 g, 5 minutes); value was determined with a DAMON/IEC hematocrit reader. A densitometer was used to measure hemoglobin. Reported normal value for hematocrit was 37.9% ± 3.4% and for hemoglobin 14.9 g/dL ± 1.3 g/dL [4]. The Giemsa staining described by Akkan et al. [9] was used for these 130 samples for blood smears; samples in which there was at least one intraerythrocyte hemoparasite were considered positive.

### 2.4. Extraction, Polymerase Chain Reaction (PCR), and Sequencing

The Promega Wizard Genomic DNA Purification Kit (Madison, Wisconsin, USA) was used to extract the DNA from the 130 blood samples. Nested PCR as described by Battsetseg et al. [14] was carried out. Primers BC48F1 and BC48R3 were used for* B. caballi* for the first PCR and BC48F11 and BC48R31 for the second PCR. The mix for the first reaction was 12.5 *μ*L and contained 6.5 *μ*L of Dream Taq PCR Master Mix 2X (Fermentas), 0.5 *μ*L of each primer (100 pmol/*μ*L), 1 *μ*L of DNA (1.9–2.6 *μ*g), and 4.5 *μ*L of nuclease-free water (Fermentas), and the amplification conditions were 4 minutes of initial denaturalization at 96°C, 40 repeated denaturing cycles (94°C for 1 minute), aligning (56°C for 2 minutes), extension (72°C for 2 minutes), and 5 minutes of final extension at 72°C. For the second PCR the conditions were the same, except that the aligning temperature was 50°C for 2 minutes, and 1 *μ*L of the first PCR was used for the reaction. DNA extracted from a positive horse, donated by the University of Veterinary Medicine Hannover, Germany, was used as a positive control. Nuclease-free water (Fermentas) was used as a negative control.

Primers EMA-5 and EMA-6 (first PCR) and EMA-7 and EMA-8 (second PCR) were used for* T. equi*. The mix for the first PCR reaction was the same as the one for the PCR of* B. caballi*. The conditions were 10 minutes of initial denaturalization at 95°C, 40 repeated denaturing cycles (94°C for 1 minute), aligning (60°C for 1 minute), extension (72°C for 1 minute), and 5 minutes of final extension at 72°C. For the second PCR the conditions were the same as those described. For the reaction, 1 *μ*L of product from the first PCR was used. DNA extracted from a positive horse, donated by the School of Veterinary Medicine and Zootechnics of the University of Sao Paulo, Brazil, was used as a positive control. Nuclease-free water (Fermentas) was used as a negative control.

The products obtained were subjected to electrophoresis (100 volts, 45 minutes) in agarose gels at 2% in TBE with GelRed. Samples that showed products with a size of 430 bp (*B. caballi*) and 218 bb (*T. equi*) were considered positive.

An amplified product of a blood sample that gave positive results for* B. caballi* and another one that tested positive for* T. equi* were sent to Macrogen, Korea, to be sequenced. Sequences were compared with others reported in GenBank, using MEGA 5.05 software and the BLAST program (National Center for Biotechnology Information, Bethesda, Maryland, USA).

### 2.5. Competitive Immunoassays (c-ELISA)

The 130 selected sera were analyzed with competitive ELISA (VMRD Inc., Pullman, Washington, United States) to detect antibodies against* B. caballi* and* T. equi*, following recommended protocols. Sera that showed percentages of inhibition greater than or equal to 40% were considered positive.

### 2.6. Statistical Analysis

Absolute and relative frequency analyses were carried out on the characteristics of the 285 horses present in the four communities. In addition, the global percentage of positive samples was calculated for both PCR and c-ELISA, as well as the specific percentage per indigenous community studied. Finally the factors associated with the presence of each protozoan were determined (positive samples to PCR) using Poisson regression. The statistical analysis was carried out using the program STATA I/C 13 (StataCorp, USA).

## 3. Results

A total of 240 indigenous individuals came to the four locations, with 285 horses. Body condition was found to be poor in 84 (29.5%) and regular in 112 (39.3%) horses; likewise, coat condition was poor (39, 13.7%) or regular (179, 62.8%). No significant differences were determined between communities (Table 1).

Hematocrit and hemoglobin average values were 19.0% and 10.65 g/dL, respectively. A total of 26 (20.0%) and 60 (46.2%) horses were positive in PCR for* B. caballi* and* T. equi*, respectively; 10 (7.7%) showed mixed infections. Positive blood samples for* B. caballi* and* T. equi* showed 98.6% (355/360 bp) homology with* B. caballi* (JN217099) and 99.1% (231/233 bp) with* T. equi* (AF261824), respectively. Erythrocyte inclusions were observed in a total of 32 (24.6%) horses. From these positive results, the PCR only confirmed 19 (59.3%) as positive. In 98 (75.4%) samples, inclusions were not found, but 57 (43.8%) were positive in PCR analysis for one of the two agents.

A total of 124 (95.4%) of the 130 horses analyzed with c-ELISA were found to have antibodies against one of the two agents: 90 (69.2%) against* B. caballi* and 115 (88.5%) against* T. equi*; of these, 81 (62.3%) had antibodies against the two agents. For* B. caballi*, 25 (19.2%) samples were positive using c-ELISA and PCR and 39 (30.0%) negative in both tests. In 50.0% (65) of samples identified as seropositive, the presence of* B. caballi* was not confirmed through PCR, while only one seronegative sample yielded positive results using PCR. For* T. equi*, 53 (40.8%) samples were positive and 8 (6.2%) negative using the two techniques. In 47.7% (62) of seropositive samples presence of* T. equi* was not confirmed using PCR, while seven (5.4%) seronegative samples reacted PCR positive.  Table 2 shows the distribution of horses found positive using PCR; significant differences were observed only in the PCR for* T. equi* between Alto Pacuare and the other communities.

In the Poisson regression analysis, as well as in logistic regression analysis, to determine epidemiological association with results of c-ELISA and PCR, respectively, factors associated with any of the conditions studied were not identified.

## 4. Discussion

The clinical exam and survey showed that most horses (196/285, 68.8%) had a body condition between regular and poor, which may be due to the low quality of the food they consume or the lack of programs to treat them for parasites. Owners indicated that they fed their horses with grass. Only 12.3% treated them for parasites a year ago, while the remaining (87.7%) did not treat them for parasites. Therefore it cannot be ruled out that gastrointestinal parasitism could have contributed to the anorexia and emaciation that were determined [3]. In addition, a high level of tick infestation was observed (99.3%) in the horses from all locations. Since ticks are not just vectors for piroplasmosis but also consume blood, this may have affected the low hematocrit and hemoglobin values found in the present study [8].

Although seroprevalence from* B. caballi *and* T. equi* was determined only from 130 horses in indigenous communities, selected from the most affected to least affected, it was much higher (69.2% and 88.5%, resp.) than that reported in thoroughbred stabled equines in Costa Rica (19% and 38%, resp.) [22]. Additionally, these agents had not been detected before through molecular techniques in our country. PCR determined the presence of these agents in 66.2% (*B. caballi* 20.0%,* T. equi *46.2%) of the horses analyzed, findings that were confirmed with sequencing. The high presence of hemoparasites detected in the horses through PCR analysis is consistent with data reported in other Latin American countries [3, 4, 24], although it must be taken into consideration that the most affected horses were chosen and analyzed in the present study.

Some studies suggest a higher infection rate of* B. caballi* than with* T. equi* [5, 25] while others [26] found greater seroprevalence of* T. equi *than of* B. equi*, which is consistent with previous findings of Jiménez et al. [22] in thoroughbred stabled equines of the Central Valley of our country and with our findings. The laboratory results suggest that the two agents are endemic in the zones where indigenous communities are located, indicating piroplasm presence without causing serious diseases in the horses. In this respect, 7.7% of animals detected with mixed infections did not present clinical characteristics different than those of animals with simple infections [1]. Endemicity in indigenous communities is probably due to the presence of a great amount of ticks, which were found on the horses and which constantly transmit piroplasms from infected to susceptible animals. This may also represent a potential risk for indigenous individuals, because most of them live in close proximity to their animals and may acquire babesial agents through ticks [1, 27, 28].

Horses with positive PCR and ELISA results represent animals with chronic infections. A greater percentage of horses persistently infected with* T. equi *than with* B. caballi* were observed, which is in agreement with other reports [6, 17, 29]. Horses with positive PCR and negative ELISA results represent animals with recent infections. In addition, in this study, more animals were detected with early* T. equi *than with* B. caballi *infections. Finally, only approximately 50% of the animals had antibodies, implying that these horses recently overcame the infection [29]; this however should be further investigated [6]. Special attention should be given to the fact that a greater presence of* T. equi *was detected in indigenous communities, because this agent is considered more pathogenic and has been related to babesial infections in humans due to its taxonomic closeness to* Theileria microti *(formerly* Babesia microti*), which causes most clinical cases in humans. Although* B. caballi *and* T. equi* (named as* B. equi*) serologically positive human cases have been reported [1, 27, 28], the OIE concludes that human babesiosis is a disease that has not been sufficiently studied, and a risk for human populations cannot be ruled out [2].

Blood smears were shown to have a low sensitivity (24.6%) to detect hemoparasites, although they are a simple, inexpensive, and useful tool to diagnose the disease in the field [2, 6].

The results obtained in the different communities did not show significant differences, which is probably due to similarities of climate and ecological factors, as well as in the ways in which indigenous individuals handle their animals, which were very similar in the four communities [30], and the fact that most affected horses were chosen. Differences in sex, race, or ages of the horses did not appear to influence the chances for infection with* B. caballi* and* T. equi*, which is consistent with findings by Kakoma and Mehlhorn [31] but contrasts with findings of Jiménez et al. [22], who reported that thoroughbred stabled equines in the Central Valley with access to pastures and older than three years were more likely to be seropositive to* B. caballi* and* T. equi*, respectively. Significant effects of the variables analyzed in the present study between groups of horses with positive and negative results to* B. caballi* and* T. equi *were not observed.Body and coat condition were not good in most horses; it was also found that they showed low hematocrit and hemoglobin values, and all animals had ticks. Although not treating the horses for parasites is considered to be an important factor in determining the occurrence of equine piroplasmosis, since it favors the presence of ticks that act as vectors of the disease, this did not turn out to be statistically significant either, given that most animals had not been dewormed [2, 12].

## 5. Conclusions

The ability to diagnose infected animals with certainty is important to be able to provide correct treatment and to prevent further hemoparasite transmission to susceptible animals. The present study proved that using two diagnostic techniques, one direct (PCR) and the other indirect (c-ELISA), is advisable to discern between animals pursuing an active or persistent infection. It is recommended that indigenous communities be informed of the risk to horses and inhabitants of contracting an infection with babesial agents from the ticks that infest their animals, and integral tick control should be promoted in the locations studied, seeking to prevent the spread of disease between horses and its transmission to indigenous individuals.

## Figures and Tables

**Table 1:** Results of the clinical exam of equines (*n* = 285) in four indigenous communities in Costa Rica.

Variable	Variable level	*n*	%	CI 95%	
				LL	UL
Community	Vereh	66	23.2	18.3	28.1
	Paso Marcos	80	28.1	22.9	33.4
	Alto Pacuare	72	25.3	20.3	30.3
	Amubre	67	23.5	18.6	24.3

Age	Young	110	38.6	32.9	44.3
	Adult	145	50.9	45.1	56.7
	ND	30	10.5	7.0	14.1

Body condition	Poor-regular	196	68.8	63.4	74.2
	Good	77	27.0	21.9	32.2
	ND	12	4.2	1.9	6.5

Fur condition	Poor-regular	218	76.5	71.6	81.4
	Good	61	21.4	16.6	26.2
	ND	6	2.1	0.4	3.8

Mucous membranes	Pale-jaundiced	54	18.9	14.4	23.5
	Pink	212	74.4	69.3	79.5
	ND	19	6.7	3.8	9.6

Number of ticks	≥50	149	52.3	46.5	58.1
	<50	134	47.0	41.2	52.8
	ND	2	0.7	−0.3	1.7

ND: no data available; CI 95%: confidence interval 95%; LL: lower limit; UL: upper limit.

**Table 2:** Absolute frequency and percentage of horses testing positive for *B*. *caballi *and *T*. *equi* using nested PCR and c-ELISA in four indigenous communities of Costa Rica (*n* = 285).

Community (*N*)	*n*	* B*. *caballi*		*T*. *equi*		*B*. *caballi* and *T*. *equi*	
		*n+* (%)		*n+* (%)		*n+* (%)	
		PCR	ELISA	PCR	ELISA	PCR	ELISA
Vereh (66)	30	8 (26.7)	20 (66.7)	12 (40.0)^a^	26 (86.7)	2 (6.7)	17 (56.7)
Amubre (66)	30	10 (33.3)	20 (66.7)	10 (33.3)^a^	28 (93.3)	4 (13.3)	20 (66.7)
Paso Marcos (80)	37	6 (16.2)	22 (59.5)	9 (24.3)^a^	35 (94.6)	2 (5.4)	22 (59.5)
Alto Pacuare (73)	33	2 (6.1)	28 (84.8)	29 (87.9)^b^	26 (78.8)	2 (6.1)	22 (66.7)
Total	130	26 (20.0)	90 (69.2)	60 (46.2)	115 (88.5)	10 (7.7)	81 (62.3)

*N* = total horses sampled; *n* = total horses analyzed; *n*+ = total positive by test.

The percentage (%) is calculated with respect to the total of animals analyzed by community. Superscripts indicate a statistical difference at the 0.05 significance level within the column, between rows.
